# Exosomes in Sepsis

**DOI:** 10.3389/fimmu.2020.02140

**Published:** 2020-09-09

**Authors:** Atsushi Murao, Max Brenner, Monowar Aziz, Ping Wang

**Affiliations:** ^1^Center for Immunology and Inflammation, The Feinstein Institutes for Medical Research, Manhasset, NY, United States; ^2^Department of Molecular Medicine, Zucker School of Medicine at Hofstra/Northwell, Manhasset, NY, United States; ^3^Department of Surgery, Zucker School of Medicine at Hofstra/Northwell, Manhasset, NY, United States

**Keywords:** sepsis, exosome, DAMP, toll-like receptor, neutrophil

## Abstract

Sepsis is a severe state of infection with high mortality. Pathogen-associated molecular patterns and damage-associated molecular patterns (DAMPs) initiate dysregulated systemic inflammation upon binding to pattern recognition receptors. Exosomes are endosome-derived vesicles, which carry proteins, lipids and nucleic acids, and facilitate intercellular communications. Studies have shown altered contents and function of exosomes during sepsis. In sepsis, exosomes carry increased levels of cytokines and DAMPs to induce inflammation. Exosomal DAMPs include, but are not limited to, high mobility group box 1, heat shock proteins, histones, adenosine triphosphate, and extracellular RNA. Exosomes released during sepsis have impact on multiple organs, including the lungs, kidneys, liver, cardiovascular system, and central nervous system. Here, we review the mechanisms of inflammation caused by exosomes, and their contribution to multiple organ dysfunction in sepsis.

## Introduction

Sepsis is systemic inflammation that occurs due to dysregulated host immune response to infection (1). Sepsis affects approximately 30 million people worldwide leading to 6 million deaths every year (2). Innate immunity plays a central role in sepsis. The two major types of molecules which induce sepsis are pathogen-associated molecular patterns (PAMPs) and damage-associated molecular patterns (DAMPs). PAMPs are molecules derived from microorganisms, typically represented by lipopolysaccharide (LPS) (3). DAMPs, on the other hand, are molecules released from stressed or dying cells (3). PAMPs and DAMPs induce inflammation upon binding to pattern recognition receptors (PRRs) (3). Systemic inflammation induced by PAMPs and DAMPs cause multiple organ dysfunction, which is the main cause of death due to sepsis (4). Despite decades of research, most of the clinical trials in sepsis have failed (5). A better understanding of sepsis pathology is essential to break through the current situation and significantly improve sepsis outcomes.

Exosomes are ∼40–150 nm endosome-derived vesicles released from cells (6). To form and release of exosomes, at first, intraluminal vesicles (ILV) are formed by inward budding of endosomes to form multivesicular bodies (MVBs), and then MVBs fuse with plasma membrane to release exosomes into extracellular space by exocytosis (6). Exosomes facilitate intercellular communication by routinely transferring functional protein, lipid and nucleic acid biomolecules from cell to cell (6, 7). Another type of vesicles are ectosomes, which are generated by outward budding of the plasma membrane and have a size of ∼50 nm to 1 mm (6). Extracellular vehicles (EVs) usually represent a broader definition, which includes both two types of vesicles (6). Since there are still technical difficulties in isolating and purifying exosomes, especially in the sense of distinguishing them from other EVs, the International Society for Extracellular Vesicles (ISEV) encourages to use “EVs” unless authors can provide enough evidence for the identification of the vesicles they worked on within their own experimental system (8). In fact, most exosomes or EVs in their literature are isolated using the same strategy (i.e., ultracentrifugation at >100,000 × *g*). Therefore, in this review we also included articles on EVs. On the other hand, we have excluded the literatures using lower-speed centrifugation, which isolates larger vesicles, even when they were called exosomes or EVs in the literature (9). Sometimes, the original articles use the terms “microvesicles” or “microparticles” instead of EVs. However, here we mark them either exosomes or EVs, based on their method of isolation, to avoid any confusion.

The clinical importance of exosomes in sepsis, especially pertaining to their use as biomarkers and mediators, has already been reviewed extensively (10). Therefore, here we mainly focus on the experimental models and the mechanisms revealed in basic research. Although exosomes can be used for drug delivery, we mainly summarize the characteristics of naturally occurring exosomes as opposed to those artificially generated. Exosomes derived from some cell types, such as mesenchymal stem cells (MSC), and some biological contents of exosomes, such as a part of miRNAs, have been shown to have therapeutic potential, but here we mainly focus on the mechanisms of inflammation and sepsis development (6). Even though not all the cited publications had a primary focus on sepsis, we incorporated all identified articles that were related with sepsis pathology.

Here we briefly summarize the overall impact of exosomes in sepsis, followed by the detailed review of the studies, which revealed their contents, the interacting signaling pathways, and the impact on different cell types and organs.

## Exosomes in Sepsis

Exosomes play a significant role in sepsis, since they can induce inflammation by activating cells with their contents (6). The number of exosomes was elevated in the sera of mice after the injection of LPS, a major bacterial component which induces endotoxemia – a syndrome that recapitulates most aspects of sepsis (11). *In vitro* experiments also showed that the amount of exosomes was increased in the media of LPS-challenged cells (12). These experiments indicate the possibilities of increased release and decreased uptake of exosomes during sepsis. Consistent with the increase number of neutrophils in sepsis, neutrophil-derived EVs dramatically increased in the blood of mice subjected to cecal ligation and puncture (CLP), a commonly used animal model of sepsis due to suppurated peritonitis (13). The overall contribution of exosomes to sepsis was studied using GW4869, which inhibits exosome generation. Treatment with GW4869 significantly improved the survival of mice subjected to LPS-injection or CLP, suggesting exosomes might play important roles in sepsis (14).

## Contents of Exosomes and Signaling Pathways to Induce Inflammation

The contents of exosomes vary among different cell types of origin, pathological condition, and environment. Besides sepsis, many studies revealed the heterogeneity of exosomes in terms of their markers and also their function. For example, the proteome of exosomes of breast cancer was able to show whether the cell of origin was epithelial like or mesenchymal like (6). Another example is exosomes from antigen-presenting cells containing MHC-II with the antigenic peptide together with co-stimulatory signals, enable direct antigen presentation to T cells, followed by T cells activation (6). In sepsis, as well, exosomes characteristics and contents may differ compared to the healthy state.

### Cytokines

Cytokines are small proteins which induce cellular signaling. Cytokines are critical factors in sepsis, whose pathogenesis is typically described as a “cytokine storm” (15). Sepsis patients show increased levels of various cytokines, both proinflammatory and immunosuppressive, locally, and systemically (15, 16). Protein array analysis of exosomes from septic mice showed that some of the pro-inflammatory cytokines, such as IL-1β, IL-2, IL-6, and TNF-α, started to augment in the early phase followed by other pro-inflammatory cytokines, including IL-12, IL-15, IL-17, and IFN-γ, in the late phase (11). Consistent with sepsis pathology, anti-inflammatory cytokines, IL-4 and IL-10, contained in exosomes of sepsis mice increased in the late phase (11). Moreover, GW4869, exosome inhibitor, significantly attenuated the levels of systemic cytokines of mice subjected to LPS-injection or CLP (14). Exosomal cytokines were suggested to be biologically active upon interacting with cells, even when they were encapsulated in the interior of the exosomes (17).

### TLR Signaling

Toll-like receptors (TLRs) are arguably the most important and well-studied PRRs, which initiate innate immunity (18). The most typical mechanism to describe the initiation of sepsis is the activation of TLR4 by LPS, which is a major components of the outer membrane of gram-negative bacteria (18). The activation of TLR4 and its adaptor protein, MyD88, induces the downstream signaling that leads to NF-κB activation and results in cytokine production (18). Other types of TLRs, such as TLR2, TLR7, and TLR9, have also been implicated in sepsis pathogenesis (19). The involvement of TLRs in the release of inflammatory exosomes in sepsis was suggested by the cells’ responsiveness to LPS, and further demonstrated by studies with DAMPs, described below. Conversely, exosomes in sepsis were able to induce TLR signaling when they were co-cultured with cells such as macrophages (20–25). Exosomes isolated from septic mice or released from cells stimulated with LPS or infected with pathogens initiated cellular signaling via TLRs, including TLR2, TLR4, and TLR7, along with MyD88, activated NF-κB, and promoted the production of cytokines and chemokines, such as IL-6, TNF-α, IL-1β, and MIP-2 (20–25). Exosomes from LPS-stimulated monocytes upregulated ICAM-1 and chemokine ligand (CCL)-2 mRNAs in endothelial cells via TLR4 and NF-κB, suggesting their potential contribution to neutrophil migration and vascular leakage (25). Furthermore, exosomes have been implicated in the transfer of TLR4 to TLR4-negative cells by studies in which TLR4 knock-out cells co-cultured with WT exosomes regained cellular responsiveness to LPS (26).

### NLR/Inflammasome Signaling

Nod like receptors (NLRs) are other important PRRs in inflammation, including sepsis (27). Among the most characterized is NLR family pyrin domain containing 3 (NLRP3) inflammasome, which is a molecular complex known to have potential to activate caspase-1 and induce IL-1β release (27). Exosomes released from LPS-treated macrophages induced NLRP3 inflammasome and caspase-1 activation of cells *in vitro* and *in vivo*, which was associated with the infiltration of macrophages and neutrophils into the tissues (28). The proteomic profiling of the exosomes showed the upregulation of exosomal proteins related with NOD-like receptor signaling pathway (28). A relevant study showed that EVs derived from Staphylococcus aureus activated NLRP3 inflammasome and caspase-1 of human macrophages, which resulted in IL-1β and IL-18 release (29). Pore forming toxins contained in EVs were critical for the NLRP3-dependent caspase-1 activation (29). Besides the role of exosomes on the activation of NLR signaling, caspase-1 in the interior of exosomes (but not on their surface) induced endothelial cell apoptosis (30). Given the fact that inflammasome plays a pivotal role in sepsis-induced inflammation and tissue injury, exosomal inflammasome may take part in fueling inflammation in sepsis.

### DAMPs

**FIGURE 1:**
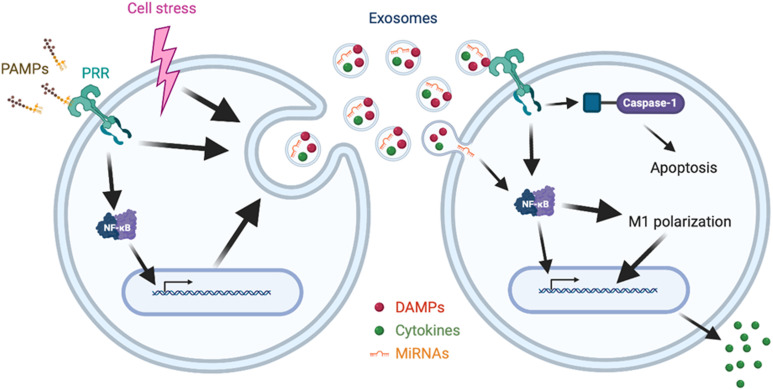
Synopsis of release and impact of exosomes in sepsis. Cellular signaling initiated by PAMPs-PRRs interaction or cell stress promotes the release of exosomes enriched with cytokines, DAMPs, and miRNAs. The released exosomes induce cellular signaling via PRRs, NF-κB or caspase-1, leading to apoptosis, M1 polarization, and cytokine release.

Damage-associated molecular patterns are endogenous molecules released upon cellular stress or tissue injury and initiate inflammatory signaling by binding to PRRs (3). DAMPs are released not only passively by necrosis, but also actively via cytoplasmic vesicles (3). Indeed, exosomes or EVs are shown to carry increased levels of DAMPs, including high mobility group box 1 (HMGB1), heat shock proteins, histones, adenosine triphosphate (ATP), and extracellular RNAs (exRNAs), in septic condition or upon LPS stimulation as described later individually. Of note, most of the preceding studies about DAMPs regarded them as contents of exosomes, and the impact of DAMPs on exosomal release and exosomal characteristics is still largely unknown. Here, we briefly describe the exosomal contents of DAMPs and their impacts on inflammation (Figure 1 and Table 1).

**TABLE 1:** Exosomal DAMPs and their impact on inflammation.

DAMPs in exosomes	Stimuli	Interacting pathway	Outcomes (production of pro-inflammatory molecules)
HMGB1	LPS (31), Pathogen (24), IL-1β (32), TNF-α (32)	TLR4 (31), Caspase-11/GSDMD (31), Autophagy deficiency (32), ER stress (33), NF-κB (24)	IL-1α (32), IL-1β (32), IL-6 (32), CCL2 (32), COX-2 (32)
HSPs	Heat shock (36), LPS (36), Pathogen (37), IL-1β (32), TNF-α (32)	Autophagy deficiency (32), ER stress (33), NF-κB (37)	TNF-α (37), IL-1α (32), IL-1β (32), IL-6 (32), CCL2 (32), COX-2 (32)
Histones	LPS (21)	TLR4 (21), ER stress (33)	TNF-α (21), IL-1β (21), IL-6 (21)
ATP	LPS (42)	P2Y11 (42)	TNF-α (42), IL-1β (42), IL-6 (42), IL-12 (42), M1 polarization (42)
exRNAs	LPS (51, 53)	TLRs (45), NLRs (45), RLRs (45), Apoptosis (46), NF-κB (52, 53), SOCS-1↓ (52, 53), SHIP1↓ (52)	MCP-1 (53), IL-1β (53), IL-6 (52, 53), TNF-α (52, 53), iNOS (53), MΦ proliferation (52), M1 polarization (53)

*HMGB1, high mobility group box 1; TLR, Toll-like receptor; GSDMD, Gasdermin D; COX-2, cyclooxygenase-2; HSPs, heat shock proteins; ER, endoplasmic reticulum; ATP, adenosine triphosphate; exRNA, extracellular RNA; SOCS, suppressors of cytokine signaling; MCP-1, monocyte chemoattractant protein 1; SHIP1, Src homology 2 (SH2) domain containing inositol polyphosphate 5-phosphatase 1.*

#### HMGB1

High mobility group box 1 is a nuclear protein which regulates gene expression and chromatin architecture intracellularly, but is also known as a DAMP once it is released into the extracellular space (3). Consistent with the elevated HMGB1 levels in sepsis patients, exosomes released from cells challenged with LPS or infected with pathogen contain increased levels of HMGB1 (24, 31). In addition, injection of GW4869, exosome inhibitor, or knockdown of Rab27, a GTPase required for exosome release, into LPS-challenged mice resulted in significant lower levels of HMGB1 in the plasma, suggesting the importance of exosomes as couriers of HMGB1 during sepsis (31). Several mechanisms of the release of exosomal HMGB1 from cells have been revealed so far, such as the activation of TLR4 and caspase-11/gasdermin D (GSDMD) signaling, decreased autophagy and endoplasmic reticulum (ER) stress, all of which are important cellular mechanisms of sepsis pathophysiology (31–33). HMGB1-enriched exosomes released from autophagy-deficient cells treated with IL-1β and TNF-α induced pro-inflammatory genes, including IL-1α, IL-1β, IL-6, Ccl2, and Cox2, when it was co-cultured with cells (32). Depletion of HMGB1 resulted in attenuated NF-κB activation induced by the exosomes (24). HMGB1 usually induces intracellular signaling pathways by binding to PRRs, including advanced glycation end products (RAGE), TLR2, TLR4, TLR9, and triggering receptor expressed on myeloid cells-1 (TREM-1) (3). However, it still remains to be confirmed whether exosomal HMGB1 works via similar mechanisms as free HMGB1.

#### Heat Shock Proteins

Heat shock proteins (HSPs) are molecular chaperones maintaining cellular homeostasis, but extracellular HSPs often work as DAMPs to induce inflammation (34). HSP70 is one of the major molecules found in exosomes and often used as a marker to identify exosomes (9). The clinical relevance of HSP70 in sepsis was indicated by its elevated levels in the sera of septic patients, which correlated with oxidative damage (35). A couple of *in vitro* experiments showed that the levels of exosomal HSP70 were increased after heat shock and further augmented by LPS stimulation, as well as by ER stress and mycobacterial infection (33, 36, 37). Similar to extracellular HSP70, exosomal HSP70 has the potential to become pro-inflammatory as it induced TNF-α release from macrophages via NF-κB activation (37). HSP90 also works as a DAMP by inducing inflammatory signaling or prolonging neutrophil survival, and its inhibition is shown to attenuate organ dysfunction and improve survival in experimental model of sepsis (38, 39). Exosomal HSP90 was shown to be elevated under autophagy deficiency with IL-1β and TNF-α treatment, and was associated with the induction of pro-inflammatory genes, including IL-1α, IL-1β, IL-6, Ccl2, and Cox2, of its affecting cells (32).

#### Histones

Histones are proteins which are components of chromatin. Histone families H2A, H2B, H3, and H4 form nucleosome and regulate gene transcription and chromatin compaction. Extracellular histones can bind to TLRs and act as DAMPs (3). Histones are released to extracellular environment during sepsis due to cellular stress and are related with its severity, including multiple organ dysfunction, coagulopathy and even mortality (40). Histones are also major components of neutrophil extracellular traps (NETs), which trap pathogens but can also result in tissue damage to the host (3). Consistent with sepsis pathology, histone levels of EVs were elevated by LPS stimulation and ER stress *in vitro* (21, 33). Histones were present on the outer surface of exosomes, thus were suggested to interact with TLR4 directly. Indeed, both anti-histone antibody and trypsin, which was used to remove histones on the surface of exosomes, significantly reduced the expression of pro-inflammatory cytokines, including TNF-α, IL-1β, and IL-6 of the treating cells in a TLR4-dependent manner (21).

#### ATP

Adenosine triphosphate is a nucleotide which plays a pivotal role in cellular metabolism. Extracellular ATP has potential to induce inflammation by binding to P2X receptors (P2XR), followed by the activation of the p38 MAPK signaling pathway (3). Excessive ATP in the serum of septic patients results in dysregulated neutrophil activity by interfering with the autocrine purinergic signaling system that regulates neutrophil function (41). ATP was shown to be released from macrophages by LPS stimulation via exocytosis, and ATP-enriched vesicles were found in cytosol of those cells, indicating EV-dependent pathway of ATP release in sepsis. ATP released via exocytosis were suggested to play a role in the production of cytokines, including IL-1β, IL-6, IL-12, and TNF-α, and M1 polarization of macrophages through P2Y11 receptor *in vitro* and *in vivo* (42).

#### Extracellular RNAs

While a number of extracellular RNAs or exRNAs with different function have been discovered, a part of them have potential to induce inflammatory response and act as DAMPs in sepsis (43). In general, exosomes are important couriers of exRNAs. Exosomes encapsulate exRNAs and thus protect them from degradation by RNAases in the biological fluid e.g., blood, urine, saliva, cerebrospinal fluid, breast milk, and follicular fluid (44). Exosomal exRNAs are recognized by PRRs, such as TLRs, NLRs, and RIG-I-like receptors (RLRs) of their interacting cells and initiate immune response (45). A recent study showed that exosomes released from apoptotic endothelial cells contained enormous amount of diversified viral-like RNAs, which have potential to activate PRRs (46).

Exosomes are abundant in microRNAs (miRNAs), small non-coding RNAs which regulate gene expression (44, 47). Dysregulated miRNAs in sepsis patients include, but not limited to, miR-25, miR-130b-3p, miR-133a, miR-146, miR-150, and miR-223 (48, 49). MiR-146 and miR-155 are well-characterized miRNAs induced by proinflammatory stimuli such as LPS and linked to TNF-α production (50). After both *in vitro* and *in vivo* LPS stimulation, exosomes contained increased amount of miR-146a and miR-155 (51). Exosomal miR-155 induced IL-6 and TNF-α production via NF-kB activation by targeting suppressor of cytokine signaling-1 (SOCS-1), and promoted macrophage proliferation by targeting SHIP1 (52). Exosomal miR-19b-3p was also increased by LPS stimulation and promoted M1 polarization and cytokine production (53). In the serum of CLP mice, exosomes showed elevated levels of miR-16, miR-17, miR-20a, miR-20b, miR-26a, and miR-26b, however their effects were not tested in the study (54).

## Impact of Exosomes on Immune Cells

Exosomes have an impact on different types of immune cells during sepsis. In sepsis, exosomes affect macrophages to induce NF-κB activation, cytokine production, such as IL-1β, IL-6, IL-12, and TNF-α, and M1 polarization as we aforementioned (20–23, 42, 53). Regulatory T cell-derived exosomes increased IL-10 and decreased IL-6 production of dendritic cells upon LPS stimulation, indicating their contribution to immunosuppression in sepsis (55). Lymphocytes are also affected by extracellular exosomes. Septic mice release exosomes, which promote Th1 and Th2 differentiation and induce the proliferation and migration of lymphocytes (11). A study showed that exosomes from LPS-treated dendritic cells endowed B cells with the ability to activate naive T cells *in vitro* and primed naive T cells via MHC class II and ICAM-1 *in vivo* (56). Another study showed that exosomes from LPS-challenged dendritic cells encountered with activated T cells were enriched in miR-155, HLA-I and ICAM-1, and were able to activate peptide-specific CD8^+^ T-cells (57).

In the above sections while describing the impact of exosomal contents in inducing inflammation in immune-reactive cells, macrophages, and lymphocytes were mainly emphasized. However, in sepsis neutrophils are one of the major leukocytes to play first line of defense as well as cause exaggerated inflammation and tissue injury when they are aberrantly activated (3). Upon activation, neutrophils expel their chromatin contents decorated with citrulinated histone H3, MPO, and DAMPs, which are collectively called NETs (3). Although NETs may trap and kill bacteria, excessive NET formation in sepsis cause tissue damage (3). DAMPs like eCIRP directly induces NETosis in sepsis in lungs and blood (58, 59). This implies that exosomes may induce NET formation as they contain DAMPs and other stimulants of NETs (Table 1). Further studies are awaited to confirm this implication. Besides DAMPs, exosomal miR-146a released from macrophages challenged with oxidized low-density lipoprotein (oxLDL), a molecular complex shown to be involved in sepsis pathology, promoted NET formation via inducing oxidative stress (60, 61).

## Impact of Exosomes on Organ Systems

Organ dysfunction is the major cause of death during sepsis (4). Organ dysfunction in sepsis has been particularly highlighted after it was explicitly included in the current SEPSIS-3 diagnostic criteria requiring a ≥2-point increase in the Sequential Organ Failure Assessment (SOFA) Score (1). Exosomes can promote the dysfunction of multiple organs in sepsis. Here, we described about exosomes in sepsis and their impact on organ dysfunction (Table 2).

**TABLE 2:** Exosomes in sepsis and their impact on organ dysfunction.

Organs	Exosomal contents	Outcomes (*ex vivo*)	Outcomes (*in vivo*)
Lungs	Caspase-1 (30), MiR-155 (52)	Apoptosis (30)	TNF-α (52), IL-6 (52), MPO (52), MΦ infiltration (52), M1 polarization (52)
Kidneys	MiR-19b-3p (53), GPRC5B (66)	NF-κB (53), SOCS-1↓(53), ERK1/2 (66), MCP-1 (53), IL-1β (53), IL-6 (53), TNF-α (53), iNOS (53), M1 polarization (53), Tubulogenesis (66)	MCP-1 (53), IL-6 (53), MΦ infiltration (53), Tubulointerstitial inflammation (53)
Liver	HMGB1 (32), HSP90 (32), MiR-155 (70), MiR-103-3p (72)	NLRP3 (28), Caspase-1 (28), IL-1α (32), IL-1β (32), IL-6 (32), CCL2,(32) COX-2 (32), α-SMA (72), TGF-β (72), Col1a1 (72), KLF4↓ (72)	NLRP3 (28), Caspase-1 (28), MΦ and neutrophil infiltration (28), AST (28), ALT (28), LDH (28)
Cardiovascular System	NADPH (76), ROS (76, 79), RNS (76, 77, 79)	RNS (77), Myocardial dysfunction (77), Caspase-3 (79), Apoptosis (79)	Cardiac dysfunction (14) (Ejection fraction↓, Fractional shortening↓)
CNS	MiR-146a (51, 81), MiR-155 (51)	NF-κB (51), TNF-α (51), IL-1β (51), IL-6 (51), Nitric oxide (51)	NF-κB (51), TNF-α (51, 81), IL-1β (51), IL-6 (51, 81), Nitric oxide (51)

*Abbreviations: MiR, microRNA; MPO, myeloperoxidase; GPRC5B, GPCR class C group 5 member B; SOCS, suppressors of cytokine signaling; MCP-1, monocyte chemoattractant protein 1; iNOS, Inducible nitric oxide synthase; HMGB1, high mobility group box 1; HSP90, heat shock protein 90; NLRP3, nucleotide-binding domain leucine-rich repeat (NLR) and pyrin domain containing receptor 3; COX-2, cyclooxygenase-2; α-SMA, α*-*Smooth muscle actin; TGF-β, transforming growth factor-β; KLF4, Kruppel*-*like factor 4; AST, aspartate aminotransferase; ALT, alanine aminotransferase; LDH, lactate dehydrogenase; NADPH, nicotinamide adenine dinucleotide phosphate; ROS, reactive oxygen species; RNS, reactive nitrogen species.*

### Lungs

Lung is one of the most vulnerable organs to systemic inflammation (62). Therefore, septic patients are often accompanied by acute lung injury (ALI) or acute respiratory distress syndrome (ARDS), which further increases the sepsis-associated mortality (62). In sepsis, circulating inflammatory molecules damage the alveolar-capillary barrier to induce the influx of pulmonary edema fluid and lung injury (62). Exosomes were increased in the bronchoalveolar lavage fluid (BALF) of infectious and non-infectious ALI mice (63). Caspase-1 encapsulated in exosomes released from LPS-challenged macrophages induced lung endothelial cell apoptosis, indicating their contribution to the disruption of the alveolar-capillary barrier (30). The injection of exosomes isolated from the serum of LPS-challenged mice increased the number of total and M1 macrophages and the levels of TNF-α and IL-6 in the lungs, which was associated with the increase of miR-155 (52). Although ARDS has attracted clinical attention for decades, none of the proposed therapies for ARDS including sivelestat sodium, neutrophil elastase inhibitor, have yet to show its clinical effect sufficiently (64). We might have overlooked the significance of exosomes as they cause inflammatory response and cell death in lungs independent of neutrophil activity (30, 52).

### Kidneys

Acute kidney injury (AKI) is a common complication of sepsis and is associated with increased morbidity and mortality (65). PAMPs and DAMPs activate PRRs of immune cells, endothelial cells, and tubular epithelial cells to induce cytokine production and cause inflammation in kidney during sepsis (65). MiRNA-19b-3p derived from tubular epithelial cells of LPS-induced AKI mice promoted M1 macrophage activation, which was associated with the activation of NF-κB and the upregulation of MCP-1, IL-1β, IL-6, TNF-α, and iNOS, by suppressing SOCS-1 *in vitro*, and induced the upregulation of MCP-1 and IL-6, macrophage infiltration, and tubulointerstitial inflammation of kidney *in vivo* (53). Exosomes of patients with AKI, not only limited to sepsis, carried increased GPRC5B, which promoted extracellular signal-regulated kinase 1/2 (ERK1/2) activation and tubulogenesis, a mechanism which has potential to attenuate AKI (66). Renal replacement therapy has been proposed in sepsis not only to support renal function but also to remove cytokines in the circulation to improve patient’s outcomes and maintenance of the kidney function, however its effect seems to be limited as to the later purpose according to the randomized controlled trials (67, 68). Exosomes are one of the potential targets in sepsis-induced AKI considering their contribution to the organ damage.

### Liver

Sepsis causes liver dysfunction typically indicated by increased bilirubin concentration and prolonged prothrombin time (69). Kupffer cells, neutrophils, hepatocytes and liver sinusoidal endothelial cells all contribute to the hepatic response in sepsis (70). Several potential mechanisms have been reported for the contribution of exosomes to liver dysfunction. Exosomes of LPS-treated macrophages were taken up by the hepatocytes and, subsequently, induced the activation of NLRP3 inflammasome and caspase-1, the infiltration of macrophages and neutrophils, and the elevation of AST, ALT, and LDH in the serum (28). Exosomes released from autophagy-deficient cells contained DAMPs, including HMGB1 and HSP90, and increased pro-inflammatory genes, including IL-1α, IL-1β, IL-6, Ccl2, and Cox2, of Kupffer cells (32). Exosomal miR-155, a micro-RNA with pro-inflammatory activity, was elevated in the liver of mice subjected to LPS and/or the TLR9 ligand cytidine-phosphate-guanosine (CpG), suggesting its contribution to liver dysfunction (71). Exosomes might also contribute to chronic liver dysfunction after sepsis, as exosomal miR-103-3p from LPS-activated macrophages targeted Krüppel-like factor 4 (KLF4) to increase α-SMA, TGF-β, and Col1a1 of hepatic stellate cells, which can contribute to liver fibrosis (72). As a whole, exosomes induce acute and chronic liver dysfunction in sepsis by inducing local inflammation and remodeling, respectively. Fresh frozen plasma transfusion and plasma exchange are performed to sepsis patients especially with liver failure (73). Exosomes have potential to predict and improve the efficacy of the treatment as a study showed exosomal characteristics are responsible for the responsiveness to plasma exchange (74).

### Cardiovascular System

Sepsis-induced cardiomyopathy is a main contributor to septic shock together with hypovolemia (75). Sepsis-induced cardiomyopathy is caused by pro-inflammatory mediators, mitochondrial dysfunction, oxidative stress, altered calcium regulation, abnormal autonomic nervous activity, and endothelial dysfunction (75). Exosomes from septic patients displayed increased levels of nicotinamide adenine dinucleotide phosphate (NADPH) oxidase activity and exhibited intrinsic production of reactive oxygen species (ROS) and reactive nitrogen species (RNS), all of them cause oxidative stress (76). A study showed exosomes from sepsis patients were enriched with nitric oxide (NO) and in turn induced myocardial NO production. Isolated heart and papillary muscle preparations exposed to those exosomes developed myocardial dysfunction, which supports their functional significance (77). Peroxynitrite was shown to be a major contributor to cytokine induced myocardial contractile failure (78). Exosomes in sepsis generated peroxynitrite while containing cytokines as we showed in the previous section (79), suggesting their synergistic contribution to cardiomyopathy in sepsis. *In vivo* experiments showed exosome inhibitor GW4869 significantly improved the cardiac function of mice subjected to LPS-injection or CLP, indicating the clinical impact of exosomes on sepsis-induced cardiomyopathy (14).

Exosomes also contribute to vascular damage in sepsis. A study showed that exosomes from sepsis patients induced apoptosis of endothelial cells and vascular smooth muscle cells as a result of increased NADPH activity of the exosomes (76). Similarly, another study showed exosomes in sepsis induced endothelial cell apoptosis via caspase-3 activation by generating superoxide, NO, and peroxynitrite (79). Taken together, exosomes link to oxidative stress closely and induce myocardial dysfunction and vascular cell apoptosis to develop septic shock.

### Central Nervous System (CNS)

Sepsis patients often present impaired consciousness not only due to shock but also due to septic encephalopathy (80). Septic encephalopathy is characterized by diffuse cerebral dysfunction caused by the systemic inflammatory response to an infection, even without direct infection in central nervous system (CNS) (80). Exosomes were increased in the cerebrospinal fluid (CSF) of mice injected with LPS peripherally, suggesting their importance for the molecular transport across blood-brain barrier (BBB) during sepsis (51). Exosomes purified from LPS-challenged mice were taken up by the microglia and astrocyts and promoted the production of pro-inflammatory cytokines, including TNF-α, IL-1β, and IL-6, *in vitro* and *in vitro*, associated with the elevated levels of miR-146a and miR-155 of the exosomes (51, 81). In addition, LPS acts on microglia to release EVs containing increased levels of TNF-α and IL-6, which might be clinically relevant as at least a portion of LPS was shown to be able to penetrate into the brain across BBB (82, 83). These studies show exosomes both in the circulation or released locally induce inflammation in the brain. Exosomes not only contribute to encephalopathy directly but also have potential to cause systemic immune dysfunction due to the collapse of neuroendocrine immune networks, which ultimately results in a vicious cycle of immunosuppression (84).

## Conclusion and Future Directions

Exosomes play pleiotropic roles in sepsis, as they carry a myriad of pro-inflammatory molecules, activate cellular signaling, and induce multiple organ dysfunction. This review demonstrated the release of exosomes, contents of exosomes, and the underlying mechanism of exosome-mediated immune cell activation and organ dysfunction in sepsis. As such, targeting excess exosome release or maintaining their homeostasis by facilitating cellular uptake could be the promising therapeutic approaches to treat sepsis. We have recently discovered eCIRP, a DAMP to exaggerate inflammation (85). Since several DAMPs were shown to be present in the exosomes of septic mice and patients, identification of eCIRP in exosomes isolated from sepsis mice or patients will be of great interest. Future studies should be focused on determining the interior and exterior molecules of exosomes and their impact on sepsis pathophysiology.

## Author Contributions

AM and MA designed, wrote, and revised the manuscript. MB critically reviewed the manuscript. PW reviewed and edited the manuscript, and conceived the idea. MA and PW supervised the project. All authors contributed to the article and approved the submitted version.

## Conflict of Interest

The authors declare that the research was conducted in the absence of any commercial or financial relationships that could be construed as a potential conflict of interest.
